# Strengthening insights into host responses to mastitis infection in ruminants by combining heterogeneous microarray data sources

**DOI:** 10.1186/1471-2164-12-225

**Published:** 2011-05-11

**Authors:** Sem Genini, Bouabid Badaoui, Gert Sclep, Stephen C Bishop, Dave Waddington, Marie-Hélène Pinard van der Laan, Christophe Klopp, Cédric Cabau, Hans-Martin Seyfert, Wolfram Petzl, Kirsty Jensen, Elizabeth J Glass, Astrid de Greeff, Hilde E Smith, Mari A Smits, Ingrid Olsaker, Guro M Boman, Giuliano Pisoni, Paolo Moroni, Bianca Castiglioni, Paola Cremonesi, Marcello Del Corvo, Eliane Foulon, Gilles Foucras, Rachel Rupp, Elisabetta Giuffra

**Affiliations:** 1 Parco Tecnologico Padano - CERSA, Via Einstein, 26900 Lodi, Italy; 2 The Roslin Institute and R(D)SVS, Division of Genetics and Genomics, Roslin, Midlothian, University of Edinburgh, EH25 9RG, UK; 3 INRA/AgroParisTech, UMR1236 Génétique et Diversité Animales, F-78352 Jouy en Josas, France; 4 INRA, Sigenae UR875 Biométrie et Intelligence Artificielle, BP 52627, F-31326 Castanet-Tolosan Cedex, France; 5 INRA, Sigenae UR83 Recherches Avicoles, F-37380 Nouzilly, France; 6 Leibniz Institute for Farm Animal Biology (FBN), Molecular Biology Research Unit, Wilhelm-Stahl-Allee 2, D-18196 Dummerstorf, Germany; 7 Clinic for Ruminants, Ludwig-Maximilians University, Munich, Germany; 8 Central Veterinary Institute of Wageningen UR, P.O. Box 65, 8200 AB, Lelystad, The Netherlands; 9 Wageningen UR Livestock Research, Animal Breeding and Genomics Centre, P.O. Box 65, 8200 AB, Lelystad, The Netherlands; 10 Department of Basic Sciences and Aquatic Medicine, Norwegian School of Veterinary Science, P.O. Box 8146 Dep, NO-0033 Oslo, Norway; 11 Università degli Studi di Milano, Department of Veterinary Pathology, Hygiene and Public Health, via Celoria 10, 20133 Milan, Italy; 12 Istituto di Biologia e Biotecnologia Agraria, Consiglio Nazionale delle Ricerche, Milan, Italy; 13 INRA-ENVT, UMR1225, Interactions Hôtes Agents Pathogènes, F-31300 Toulouse, France; 14 INRA, UR631, Station d'Amélioration Génétique des Animaux, F-31326 Castanet-Tolosan, France; 15 Department of Clinical Studies, School of Veterinary Medicine, University of Pennsylvania, Philadelphia, PA 19104, USA; 16 Quality Milk Production Services, Cornell University, Ithaca, New York, USA; 17 INRA, UMR 1313 de Génétique Animale et Biologie Intégrative, Jouy-en-Josas, France

**Keywords:** Meta-analysis, microarray analysis, mastitis infection, lipid metabolism, immune response

## Abstract

**Background:**

Gene expression profiling studies of mastitis in ruminants have provided key but fragmented knowledge for the understanding of the disease. A systematic combination of different expression profiling studies via meta-analysis techniques has the potential to test the extensibility of conclusions based on single studies. Using the program Pointillist, we performed meta-analysis of transcription-profiling data from six independent studies of infections with mammary gland pathogens, including samples from cattle challenged *in vivo *with *S. aureus*, *E. coli*, and *S. uberis*, samples from goats challenged *in vivo *with *S. aureus*, as well as cattle macrophages and ovine dendritic cells infected *in vitro *with *S. aureus*. We combined different time points from those studies, testing different responses to mastitis infection: overall (common signature), early stage, late stage, and cattle-specific.

**Results:**

Ingenuity Pathway Analysis of affected genes showed that the four meta-analysis combinations share biological functions and pathways (e.g. protein ubiquitination and polyamine regulation) which are intrinsic to the general disease response. In the overall response, pathways related to immune response and inflammation, as well as biological functions related to lipid metabolism were altered. This latter observation is consistent with the milk fat content depression commonly observed during mastitis infection. Complementarities between early and late stage responses were found, with a prominence of metabolic and stress signals in the early stage and of the immune response related to the lipid metabolism in the late stage; both mechanisms apparently modulated by few genes, including *XBP1 *and *SREBF1*.

The cattle-specific response was characterized by alteration of the immune response and by modification of lipid metabolism. Comparison of *E. coli *and *S. aureus *infections in cattle *in vivo *revealed that affected genes showing opposite regulation had the same altered biological functions and provided evidence that *E. coli *caused a stronger host response.

**Conclusions:**

This meta-analysis approach reinforces previous findings but also reveals several novel themes, including the involvement of genes, biological functions, and pathways that were not identified in individual studies. As such, it provides an interesting proof of principle for future studies combining information from diverse heterogeneous sources.

## Background

In the last decade, gene expression profiling microarrays have been widely used in animal genomics and this technique has enabled researchers to monitor, on a broad scale, the effects of pathogens on host cells and tissues, aiming to gain insight into the molecular mechanisms that are involved in the host-pathogen interactions. Mastitis is one of the most costly diseases of the dairy industry, which makes it among the major concerns for the livestock sector [1]. As a consequence, numerous gene expression studies on mastitis in different host species infected with various pathogens are publicly available. However, due to the high costs of this approach, most individual studies have been carried out on limited numbers of technical and biological replicates. Furthermore, different and improved microarray platforms have been used over time, due to the increased availability of improved microarray tools tailored to the genome sequence of most livestock species.

Meta-analysis can be used to combine or integrate the data or results of independent studies. It allows a more objective appraisal of evidence than individual studies and has been widely used to interpret contradictory results from various studies or overcome the problem of reduced statistical power in studies with small sample sizes (reviewed by [2,3]). The applicability of meta-analysis to microarrays was initially demonstrated by [4,5]. Subsequently, several different meta-analysis applications have been developed in order enable the integration of independent microarray expression studies, e.g. through the combination of effect sizes [6], the comparison of data intersections (comparative meta-profiling) [7,8], the integration of data from Affymetrix arrays through re-annotation and common pre-processing methods [9], the quantification of similarities in the literature (with an algorithm called LAMA, Literature-Aided Meta-Analysis) [10], the development of a ranking aggregation approach [11], and the application of improved and meta-analysis adapted normalization methods [12-14]. Meta-analysis methods have also been applied to characterize the properties of promoters to regulate transcription of up-regulated genes [15].

As p-values are usually available for each gene in each study, the main focus of the current meta-analysis approach was to increase the reliability of statistical evidence, by combining p-values across several, often heterogeneous, experiments. Various statistics have been suggested to combine p-values [2,4,16-19]. In particular, the meta-analysis tool chosen for this study, Pointillist [20,21], uses and extends the Fisher inverse chi-square method for p-value combination (reviewed by [22]) by calculating different weights (i.e. reliability/representativeness parameters which represent relative measures of statistical power of all datasets analysed) that are used to transform the p-values of each experiment. By doing so, Pointillist takes into consideration the various experimental design differences and the high heterogeneity of the datasets, including the use of different platforms, that has been a major hindrance to meta-analysis so far.

The large quantity of microarray data available for mastitis in ruminants provides an attractive opportunity for a meta-analysis approach. Gene expression commonalities shared across pathogens and host species may contribute to understanding the disease and its physiology, as well as pinpoint the most promising direction of research to identify effective biomarkers. Indeed, several innate immune responses, especially to pathogen-associated molecular patterns, show evolutionary conservation, thus increasing the feasibility of meta-analysis of gene expression data across species [23]. In controlled *in vitro *cultures of macrophages [24] and dendritic cells [25], a similar shared induction of common gene expression patterns in responses to a broad range of bacteria has been observed. Furthermore, previous meta-analysis results [26] showed common clusters of affected genes across larger numbers of pathogens and studies.

The aim of this project was to identify common sets of differentially expressed genes regulated by three mastitis pathogens (*S. aureus*, *S. uberis*, and *E. coli*) in three affected ruminant species (cattle, goat, and sheep). Economy-wise, these three species are by far the most important for the dairy industry. For this purpose we used the program Pointillist [20,21] and, by combining similar time points of different experiments, we created four main lists of genes differentially modulated by mastitis infection. *In vitro *experiments were treated in the same way as *in vivo *experiments as the weighting mechanism of Pointillist provided protection against potential response-dependant biases.

We then used the Ingenuity Pathways Analysis (IPA; http://www.ingenuity.com) software to retrieve the canonical pathways, biological functions and networks that were most significantly associated with the lists of affected genes. IPA is a curated database and web-based analysis system that delivers an assessment of signaling and metabolic pathways, molecular networks, as well as key biological and disease processes that are most significantly perturbed in a gene set of interest. For each meta-analysis combination tested with IPA, the five most affected canonical pathways and the five most affected biological functions belonging to the sub-group "molecular and cellular functions" are discussed in detail.

All the meta-analysis combinations highlighted a predominance of gene pathways and biological functions related to immune response and to lipid metabolism. The results show common but also combination-specific affected genes and pathways and provide new avenues for future studies.

## Results and discussion

### Combination of time points of mastitis experiments with Pointillist

Different combinations of time points from individual experiments (Table 1) were selected to represent four main categories of response to mastitis infection. These combinations were performed with Pointillist and were named: (I) overall response, (II) early stage response, (III) late stage response, and (IV) cattle-specific response (Table 2). No goat- or sheep-specific responses were studied because of the more limited number of experiments and time points for those species.

**Table 1:** Summary of the microarray datasets on mastitis infection included in meta-analysis

*Experiment # (Institution)*	*Host species (# of biological replicates)*	*Pathogen*	*Challenge system*	*Bovine cDNA microarray*	*Time after infection {time point}*	*Signs of infection*	*References*
**1A **(RI/RIBFA)	Cattle (4)	*E. coli*	Intramammary challenge. Sampled material: lobulo-alveolar mammary tissue (*in vivo*)	ARK-genomics 20 k	6 h {1}	No clinical signs and no alteration of *TLR2*, *TLR4*, and β-defensins expressions.	[27,63,64]
					12 h {2}	Mild clinical signs and small changes of *TLR2*, *TLR4*, and β-defensins expressions.	
					24 h {3}	Acute clinical signs (including increased SCC count, decreased milk yield, leukopenia, fever, udder swelling) and up-regulation of *TLR2*, *TLR4*, and β-defensins expressions	
**1B **(RI/RIBFA)	Cattle (4)	*S. aureus*	Intramammary challenge. Sampled material: lobulo-alveolar mammary tissue (*in vivo*)	ARK-genomics 20 k	6 h {4}	No clinical signs and no alteration of *TLR2*, *TLR4*, and β-defensins expressions.	[27,63,64]
					12 h {5}	No clinical signs and no alteration of *TLR2*, *TLR4*, and β-defensins expressions.	
					24 h {6}	No clinical signs and no alteration of *TLR2*, *TLR4*, and β-defensins expressions.	
**1C **(RI/RIBFA)	Cattle (4)	*S. aureus*	Intramammary challenge. Sampled material: lobulo-alveolar mammary tissue (*in vivo*)	ARK-genomics 20 k	12 h {7}	No clinical signs and no alteration of *TLR2*, *TLR4*, and β-defensins expressions.	[27,63,64]
					72 h {8}	Acute clinical signs (including increased SCC count, decreased milk yield, leukopenia, fever, udder swelling) and up-regulation of *TLR2*, *TLR4*, and β-defensins expressions	
**2 **(CVI-L)	Cattle (3)	*S. uberis*	Udder samples containing all layers including epithelia, muscle tissue and mammary gland tissue. In affected samples neutrophils were also present (*in vivo*)	ARK-genomics 20 k	36 h-72 h {9}	Culling when clear clinical signs were seen. Sample selection from various locations of control and infected mammary gland quarters based on clear microscopic and macroscopic observations	-
**3 **(NSVS)	Cattle (6)	*S. aureus*	Blood derived primary macrophage cells (*in vitro*)	ARK-genomics 17 k	2 h {10}	Few genes responding, no cell death.	-
					6 h {11}	Many genes responding, beginning signs of cell deformation and death	
**4 **(UNIMI/PTP/CNR)	Goat (3)	*S. aureus*	Leukocytes in milk (*in vivo*)	NBFGC	12 h {12}	No clinical signs and no alteration of milk.	[65,66]
					24 h {13}	Clear clinical signs (increased SCC count, decreased milk yield, fever)	
**5 **(INRA)	Sheep (8)	*S. aureus*	Bone marrow derived primary dendritic cells(*in vitro*)	ARK-genomics 17 k	3 h {14}	No cell death.	-
					8 h {15}	Clear deformation and death of dendritic cells	
**6 **(UNIMI/PTP/CNR)	Goat (10)	*S. aureus*	Leukocytes in milk (*in vivo*)	Combi-Matrix	24 h {16}	Clinical signs (increased SCC count, decreased milk yield, fever, udder swelling)	-

The experimental numbers are reported with the names of the institution where they were conducted, host species and number of replicates, pathogens, challenge systems, microarrays names, time period of observations after infection {in parenthesis the time point #, see also Table 2}, signs of infection, and corresponding references.

Note: **ARK-genomics**: centre for comparative & functional genomics, Scotland; **CNR**: Institute of Agricultural Biology and Biotechnology, National Research Council, Italy; **CVI-L**: Central Veterinary Institute of Wageningen UR, Lelystad, NL; **INRA**: Institute National de la Recherche Agronomique, France; **NBFGC**: National Bovine Functional Genomics Consortium, USA; **NSVS**: Norwegian School of Veterinary Science, Norway; **PTP**: Parco Tecnologico Padano (PTP), Italy; **RI**: Roslin Institute and R(D)SVS, University of Edinburgh (UEDIN), UK; **RIBFA**: Research Institute for the Biology of Farm Animals, Germany; **UNIMI**: Università degli Studi di Milano, Department of Veterinary Pathology, Hygiene and Public Health, Italy. Microarrays are described in the Materials and Methods section of text.

**Table 2:** Combination of experiments and time points to create the 4 main responses to mastitis infection

	Time after infection							
Experiment #	2 h	3 h	6 h	8 h	12 h	24 h	36 h-72 h	72 h
**1A**: *E. coli *in cattle (*in vivo*)			{1} I, II, IV		{2} I, IV	{3} I, III, IV		
**1B**: *S. aureus *in cattle (*in vivo*)			{4} I, IV		{5} I, IV	{6} I, II, IV		
**1C**: *S. aureus *in cattle (*in vivo*)					{7} I, II, IV			{8} I, III, IV
**2**: *S. uberis *in cattle (*in vivo*)							{9} I, IV	
**3**: *S. aureus *in cattle macrophages (*in vitro*)	{10} I, II, IV		{11} I, III, IV					
**4**: *S. aureus *in goat (*in vivo*)					{12} I, II	{13} I, III		
**5**: *S. aureus *in sheep dendritic cells (*in vitro*)		{14} I, II		{15} I, III				
**6**: *S. aureus *in goat (*in vivo*)						{16} I		

Combination of microarray data from a total of 6 different experiments and 16 different time points ({in parentheses}, see also Table 1 and text for details) to analyse 4 different responses to mastitis infection: (I) overall response, (II) early stage response, (III) late stage response, and (IV) cattle-specific response.

The combination (I) overall response included each animal species (cattle, sheep, goat) and all the time points (see Tables 1 and 2) in order to capture the heterogeneity of all datasets. In order to avoid bias towards cattle, for which more datasets were available, the list of combined p-values, or so-called "Combined Effective Significances", for each probe was obtained by a stepwise process. First, species-specific p-value lists were obtained. A single Pointillist run was applied to obtain the goat-specific (combination of time points {12}+{13}+{16}) and the sheep-specific (combination of time points {14}+{15}) p-value lists. To obtain the cattle-specific p-value list, (IV) cattle-specific response, two Pointillist processing steps were required. Firstly, the time points for each separate bovine microarray experiment, e.g. 1A (combination of time points {1}+{2}+{3}), 1B (combination of time points {4}+{5}+{6}), 1C (combination of time points {7}+{8}), 2 (time point {9}), and 3 (combination of time points {10}+{11}) were analyzed separately with an initial Pointillist run. Subsequently, the resulting p-values of each experiment were combined with a second Pointillist run. The final combined p-values for (I) overall response were obtained by combining with an additional Pointillist run the three species-specific p-value lists.

The combined p-value lists for (II) early stage and (III) late stage responses were obtained by combining the time points for which respectively "no signs" or "clear signs" of mastitis were observed. In particular, inclusion of *in vivo *time points {1}+{6}+{7}+{12} in list (II) and {3}+{8}+{13} in list (III) (Table 2), was supported by the absence or the clear presence, respectively, of clinical signs of acute mastitis such as increased SCC count, decreased milk yield, leukopenia, fever, and udder swelling (Table 1). The absence of clinical signs in time points {1}, {6}, and {7} had been confirmed by real-time PCR of indicators for acute mastitis (*TLR2*, *TLR4*, and β-defensins; [27]). The early time points {10} and {14} of the *in vitro *studies were assigned to the early stage response because minimal or no reaction or cell death was observed, while the later time points {11} and {15} were included in the late stage response because clear reaction or physiological deformation and death of the cells were observed. Time point {9} was neither included in the early stage nor in the late stage response because it was the only available time point for the pathogen *S. uberis*.

### Overall response to mastitis infection

Because we pooled microarrays of different designs, only 13,162 probes could be analyzed in combination (I) overall response. Of the 498 probes identified by Pointillist as being significantly altered (p ≤ 0.05), a total of 298 unique genes were present in the IPA knowledge database [Additional file 1]. The relative weights assigned by Pointillist to each species-specific experiment were 0.82 for cattle (experiments 1, 2, and 3), 0.08 for goat (experiments 4 and 6), and 0.09 for sheep (experiment 5). This indicates that the cattle data had greater statistical power than the goat- and sheep-specific data, which were similar in terms of statistical power.

### Affected canonical pathways

The 5 canonical pathways identified by IPA as being most significantly associated to this list of 298 genes were protein ubiquitination, acute phase response signaling, lipid antigen presentation by CD1, oncostatin M signaling, and antigen presentation pathway [Additional file 2].

The protein ubiquitination pathway has a fundamental role in a myriad of cellular processes, including cell proliferation, antigen presentation, and regulation of both innate and adaptive immune responses [28,29]). This pathway was present within the 5 most significant canonical pathways of the other 3 main gene lists [Additional file 2], confirming its role in defence against pathogens, including bacteria [30]. The acute phase response is a rapid, non-specific inflammatory response that provides protection against microorganisms, and is associated with the expression of several cytokines [31]. Furthermore, bovine acute phase response has been shown to be activated by lipopolysaccharide (LPS) [32] and by *E. coli *[33], possibly through its LPS. The lipid antigen presentation by CD1 and the antigen presentation pathways are important to the development of innate and adaptive immunity [34]. Finally, oncostatin M signaling is known to be responsible for the initiation and progression of inflammation and the acute phase response [35]. These findings suggest that the alteration of immune response and lipid metabolism are hallmarks of the response to infections causing mastitis.

### Affected biological functions

[Additional file 3] reports the complete lists of affected biological functions for all the sub-groups "Diseases and disorders", "Physiological system development and function" and "Molecular and cellular functions". The five most significant molecular and cellular functions altered during the overall response to mastitis were cell death, cellular movement, cellular growth and proliferation, cell-to-cell signaling and interaction, and lipid metabolism. The first three altered functions were among the 5 most affected in all 4 main responses [in bold in Additional file 3].

Perturbation of the lipid metabolism might affect the lipid antigen presentation by CD1 pathway [Additional file 2], which consists of a conserved family of MHC-like glycoproteins specialized in capturing lipid and glycolipid antigens for presentation to T lymphocytes [36]. A relevant correlation between lipid metabolism and mastitis infection caused by *S. uberis *in mammary tissues has indeed been reported [37]. Furthermore, lipid metabolism has been identified as one of the most altered biological functions in cows fed at different energy balance diets [38] and it has been associated with differentially regulated proteins detected in cows infected with *E. coli *and *S. aureus *[39]. Consequently, IPA was used to further dissect the main sub-functions linked to lipid metabolism. Metabolism of long chain fatty acids, accumulation of oleic acids, internalization of lipids, and uptake of fatty acids and arachidonic acid were the top 5 annotated functions related to lipid metabolism and altered during the overall response to mastitis [Additional file 4]. The affected biological functions further confirm a relevant role of the lipid metabolism during response to infections causing mastitis.

### Early stage and late stage responses to mastitis infection

Of the 20,527 probes analyzed by Pointillist for the early and late stage responses, 1,129 and 1,046, respectively, were significantly altered (p ≤ 0.05). Of these, a total of 639 and 631 unique genes, respectively, were present in the IPA knowledge database [Additional file 1].

### Affected canonical pathways

In addition to the protein ubiquitination and polyamine regulation pathways that were common for both combinations, the early stage response was characterized by pathways closely related to metabolic regulation, including hypoxia signaling, pyruvate metabolism, and endoplasmic reticulum (ER) stress [Additional file 2]. Hypoxia inducible factors are known to control innate immunity and gene expression of pro-inflammatory molecules [40], and correlations between ER stress, immune response and apoptosis have been reported [41]. Also, pyruvate accumulation caused by inhibition of lipid metabolism has indeed been shown to prompt hypoxia signaling in mastitis in cattle [37]. The significant alterations of these closely linked pathways suggests that stress signals are launched by the host cells as part of the activation of the immune response early during infection, i.e. prior to observation of clear phenotypes related to mastitis.

On the other hand, the late stage response was specifically represented by pathways directly involved in the immune response, i.e. IL-6 signaling, LXR/RXR activation and IL-10 signaling [Additional file 2]. A close relationship between polyamine regulation, in particular the sub-group spermine, and IL-10 signaling has been reported in macrophages [42]. Other studies reported an increase of IL-6 and IL-10 expression during mastitis infection [43,44]. As persistence or over-prolongation of inflammation is harmful for cells [45], the activation of the IL-10 signaling might be a beneficial mechanism adopted by the cells during this stage of mastitis infection to limit and terminate the inflammatory response.

### Affected biological functions

Cellular growth and proliferation, cell death and cellular movement were 3 of the top 5 significant molecular and cellular functions identified by IPA for both time-dependant responses [Additional file 3]. Two protein-related functions (post-translational modification and protein folding) were specific for the early stage response, while cellular functions (cellular assembly and organization, cell-to-cell signaling and interaction) were specific for the late stage response [Additional file 3].

Lipid metabolism was significantly altered during both early (p = 3.5E-04) and late stage (p = 3.1E-06) infections, although it was not among the five most significant. The altered LXR/RXR signaling pathway [Additional file 2] is known to be implicated in the regulation of the lipid metabolism [46]. Since lipid metabolism was among the top 5 affected molecular and cellular functions in the overall analysis (gene list I), the main altered sub-functions of the lipid metabolism were identified by IPA. Hydrolysis of phosphatidylinositol phosphate, phosphatidylinositol 4,5-diphosphate, and phosphtidylinositol 5-phosphate, as well as metabolism of fatty acid and lipids were the most significant affected sub-functions for the early stage response [Additional file 4]. For the late stage response on the other hand, quantity of fatty acid, oleic acid, and lipid, as well as synthesis of lipid and cholesterol were the identified top affected sub-functions. These results seem to suggest that whilst during the early stage response there might be a "general" deregulation of the lipid metabolism, during the late stage response the cells might react to the infection by synthesizing, taking up, or incorporating lipids and fatty acids.

### Relevance of the *XBP1 *gene during the early stage of infection

The lists of affected genes during the early and late stage responses were analyzed with the IPA feature "pathway building", which shows the main relationships and connections among affected genes belonging to altered canonical pathways. The two genes X-box binding protein 1 (*XBP1*) and sterol regulatory element binding transcription factor 1 (*SREBF1*) are of particular relevance in early and late stage infection, respectively. Both belong to canonical pathways that were among the 5 most affected (*XBP1 *to ER stress and *SREBF1 *to LXR/RXR activation) [Additional file 2] and, in agreement with their function as transcription factors, they were directly linked to the highest number of other affected genes [Additional file 5: Supplemental Figures S1B and S2].

*XBP1 *and the additional transcription factors *ATF4*, as well as the molecular chaperone *DNAJB3 *and the heat-shock protein gene *HSPA5*, which are key molecules of ER stress, one of the 5 most significantly affected pathways [Additional file 2], were altered during the early stage response. Comparable results have been reported in other studies in dairy cows where expressions of *ATF4*, *XBP1*, and *DNAJB3 *were altered in ER stress generated by a negative energy balance [47]. Hence, *XBP1 *might have a central role in launching stress signals in preparation for an adequate immune response during the early stage of mastitis infection, as it is also involved in cytokine production in different cell types, including macrophages [48,49]. This gene directly regulates the expression of the affected genes *COPZ1*, *DDOST*, *KDELR2*, *KDELR3*, *RPN1*, *SEC23B*, *SEC24D*, *SEC61A1*, *SRPR*, as well as genes of the proteasome and the MHC Class II complex [Additional file 5: Supplemental Figure S1B]. Indirectly, *XBP1 *is also linked to many more affected genes [Additional file 5: Supplemental Figure S1A]. In line with our results, alteration of several genes that directly interact with *XBP1 *(e.g. *COPZ1*, *DDOST*, *KDELR3*, *RPN1*, *SEC23B*, *SEC24D*, *SEC61A1*, and *SRPR*) have also been reported in fibroblasts over-expressing XBP1 [50].

### Relevance of the *SREBF1 *gene during the late stage of infection

In the late stage response, *SREBF1 *directly interacts with several affected lipogenic genes, i.e. *TRAF3IP3*, *CD36*, *SCD*, *SOD1*, *IDH1*, *THRB*, *RETN*, *PMVK*, *DBI*, *UCP2*, *HBS1*, *SC4MOL*, and *CYP27A1 *[Additional file 5: Supplemental Figure S2]. Among these, expressions of *TRAF3IP3*, *CD36*, and *SCD *were also reported to be altered during infection of cattle mammary tissues with *S. uberis *[37]. *SREBF1 *is a component of the LXR/RXR pathway, one of the 5 most affected pathways, confirming the relationship between LXR/RXR signalling and lipid metabolism. This relationship might explain the observed depression of milk fat synthesis during mastitis infection in ruminants.

### Early and late stage specific responses

In order to better understand the differences between the two different time-related responses, the (II) early stage and the (III) late stage responses were subjected to IPA analysis taking into account only the subset of affected genes differentiating the two lists. While 375 genes belonged to list (II) and not list (III) (list V early specific response), 367 genes belonged to list (III) and not list (II) (list VI late specific response) [Additional file 1].

### Affected canonical pathways

The results of the canonical pathway analysis confirmed that during early specific response there is intensification of cell metabolism (exemplified by the pyruvate and butanoate metabolism), the protein ubiquitination pathway, as well as the stress signal pathways, e.g. hypoxia in the cardiovascular system and Ataxia Telangiectasia Mutated (ATM) signaling [Additional file 2]. During the late specific, the top affected pathways (IL-6 signaling, polyamine regulation, acute phase response signaling, "role of macrophages, fibroblasts and endothelial cells in rheumatoid arthritis", and Fc receptor-mediated phagocytosis in macrophages and monocytes) indicated an intense activity of the immune response, with the possible involvement of macrophages.

### Affected biological functions

None of the top 5 molecular and cellular functions were in common between the two time-specific gene lists (V) and (VI) [Additional file 3]. Similarly to the previous analysis of gene list (II) early stage response and (III) late stage response, the early specific response genes showed molecular and cellular functions related to metabolism (carbohydrate and lipid), biochemistry and protein synthesis (post translational modification and folding), while the late specific response were mainly involved in cellular functions (movement, growth and proliferation, assembly and organization, function and maintenance), as well as cell morphology.

### Cattle-specific response to mastitis infection

Pointillist identified 669 out of 19,448 common probes that were significantly altered (p ≤ 0.05) in the cattle-specific response to mastitis. The weights given by Pointillist to experiments 1A, 1B, 1C, 2, and 3 were 0.27, 0.23, 0.28, 0.17, and 0.04, respectively, showing that the *in vitro *data set had a lower statistical power than the other data sets. Of the 669 probes, a total of 421 unique genes were present in the IPA knowledge database.

### Affected canonical pathways

Besides polyamine regulation and protein ubiquitination, the top canonical pathways characterizing the cattle-specific response were acute phase response, lipid antigen presentation by CD1 (also identified in the overall response), two highly relevant pathways for immune response, and the inositol metabolism [Additional file 2], which is involved in T-cell, B-cell, and neutrophil development and function [51]. These results indicate a link between mastitis and immune response involving T and B cells.

### Affected biological functions

In accordance with the top canonical pathway analysis, the altered molecular and cellular functions identified by IPA (i.e. antigen presentation, cell death, cell to cell interaction, and cellular growth, proliferation and movement) reflected an intensification of the immune response during cattle-specific response to mastitis infection [Additional file 3].

Alteration of the expression of genes involved in immune response, antigen presentation, apoptosis, and acute phase response have been also reported in a similar study [52].

Lipid metabolism was also significantly affected (p = 4.9E-05), although it was not included among the five most significant. Sub-functions of lipid metabolism that were altered during the cattle-specific response included uptake of arachidonic acid, metabolism of long chain fatty acid, internalization of cholesterol, transport and quantity of fatty acid [Additional file 4]. These findings further underline that lipid metabolism is tightly linked to immune response and that lipid antigen presentation might represent an interesting candidate pathway for future work to gain new insights into the host-pathogen interplay in mastitis.

### Comparison of the host expression profiles in the different experiments and time points

Next, we compared the different cattle microarray datasets, focusing on the impact of the use of different infective agents (three of the major mastitis-causing pathogens: *E. coli*, *S. aureus*, and *S. uberis*) and the patterns of gene response that they caused in the host.

**Figure 1:**
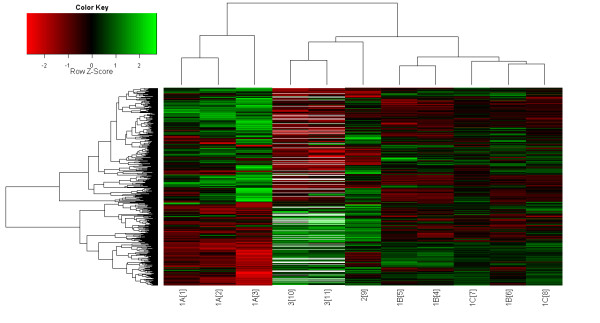
**Heat map showing cluster analysis of the microarray experiments used in the cattle-specific response to three different pathogens (*E. coli*, *S. aureus*, and *S. uberis*)**. The x-axis shows the time points {from 1 to 11} of each different cattle experiment (1A, 1B, 1C, 2, and 3; Table 2), while the y-axis displays the clustered genes. The map itself contains gene fold changes Z-score normalized over all time points. They are color coded, with red corresponding to down-regulation and green to up-regulation. White lines in experiment 3 represent missing genes not present on the microarray. The first clustering step is primarily based on experiment number (Tables 1 and 2) (experiment 1A {1-3} clustered together, 1B {4-5} clustered together, 1C {7-8} clustered together along with the 1B time point {6}, and 3 {10-11} clustered together). The final clustering steps indicated a pathogen-specific pattern as all *S. aureus *time points (along with the *S. uberis *time point {9}) clustered together, separately from the *E. coli *time points.

When clustering the expression profiles of the cattle-specific response time points (see heat map in Figure 1) the first clustering step is primarily based on experiment number (Tables 1 and 2) (experiment 1A time points {1-3} clustered together, experiment 1B time points {4-5} clustered together, experiment 1C time points {7-8} clustered together along with experiment 1B time point {6}, and experiment 3 time points {10-11} clustered together). It is not unexpected that expression profiles of different time points of the same microarray experiment cluster together. The final clustering steps indicated a pathogen-specific pattern as all *S. aureus *time points (along with the *S. uberis *time point {9}) clustered together, separately from the *E. coli *time points. No inter-laboratory or inter-array clustering was observed. For instance, the *E. coli *data (experiment 1A) did not cluster with the data from the other experiments (1B and 1C) performed in the same institution (Figure 1). This provides reassurance that the data were not significantly biased towards the experimental conditions used.

### Comparison of the strength of the host response to the 3 different pathogens

**Figure 2:**
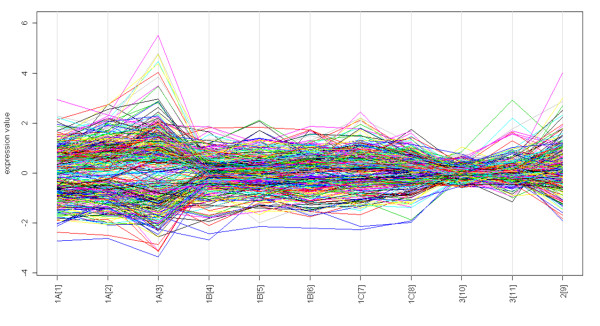
**Magnitude of fold change expression characterizing *E. coli*, *S. aureus*, and *S. uberis *infections in cattle**. The x-axis shows the time points {from 1 to 11} of each different cattle experiment (1A, 1B, 1C, 2, and 3; Table 2), while the y-axis shows the fold changes for each gene (each line). High differences are observed especially during infection with *E. coli *(1A {3}), and to a lesser extent with *S. uberis *(2 {9}).

We also compared the magnitude of fold change differences in gene expression in the cattle host caused by *E. coli*, *S. aureus*, or *S. uberis *infections with the MaSigPro package [53]. Figure 2 shows that, in general, the *E. coli *infection caused a stronger response in the host than the *S. aureus *and *S. uberis *infections. High fold change differences were induced by *E. coli*, especially at 24 h post infection (experiment 1A, time point {3}), and to a lesser extent by *S. uberis *between 36 and 72 h post infection (experiment 2, time point {9}).

Although this finding might be related to the specific experimental conditions used in the different experiments, it reflects previous observations that *E. coli *infection is very acute at 24 h, but not yet at 6 h PI [52], and that it is very acute compared to other pathogens [39,54]. Furthermore, the results suggest that *S. aureus*, but not *E. coli*, frequently causes subclinical, chronic infections of the mammary gland and hence elicits an inadequate mammary immune response [27,55].

### Comparison between meta-analysis of (IV) cattle-specific response and individual experiments

To better quantify the additional power of the proposed meta-analysis approach, we compared the list of 421 affected genes identified with the meta-analysis of (IV) cattle-specific response with the lists of affected genes (using the Benjamini-Hochberg FDR-correction [56], p ≤ 0.05) in individual experiments (i.e. experiment 1A time point {3} and experiment 2 time point {9}). The results showed that 25 affected genes were in common between the three lists, while 268, 581, and 15 genes were specific for (IV) cattle-specific response, experiment 1A time point {3}, and experiment 2 time point {9}, respectively [Additional file 5: Supplemental Figure S3 and the corresponding gene lists in Additional file 6].

Next, applying IPA on the lists of affected genes, we identified the 5 most affected canonical pathways and molecular and cellular functions of the individual experiments. The canonical pathways protein ubiquitination (p = 2.9E-09), ephrin receptor signaling (p = 1.1E-06), regulation of actin-based motility by Rho (p = 5.7E-06), actin cytoskeleton signaling (p = 3.6E-05), and germ cell-Sertoli cell junction signaling (p = 5.5E-05), as well as the molecular and cellular functions cell death, cellular growth and proliferation, cell signaling, cellular movement, and lipid metabolism were the most affected within the 745 affected genes of experiment 1A time point {3}. Canonical pathways and molecular and cellular functions in common with the five most affected identified by meta-analysis of the (IV) cattle-specific response [Additional files 2 and 3] included polyamine regulation, as well as cell death, cellular growth and proliferation, and cellular movement, respectively.

The IPA canonical pathways iCOS-iCOSL signaling in T Helper cells (p = 4.7E-04), activation of IRF by cytosolic pattern recognition receptors (p = 1.1E-03), dendritic cell maturation (p = 1.8E-03), production of nitric oxide and reactive oxygen species in macrophages (p = 1.8E-03), and communication between innate and adaptive immune cells (p = 3.0E-03), as well as the molecular and cellular functions cellular growth and proliferation, cell death, cell-to-cell signaling and interaction, cellular function and maintenance, and gene expression were the most affected within the 55 genes of experiment 2 time point {9}. None of the canonical pathways were in common with the most affected of the meta-analysis of the (IV) cattle-specific response; whereas cell death, cellular growth and proliferation, and cell-to-cell signaling and interaction were common molecular and cellular functions.

The retrieval of common molecular and cellular functions and/or pathways by the two approaches (meta-analysis vs. individual experiments) confirms the statistical power of the meta-analysis and its complementary to the FDR correction with regard to the pruning of false positives. Furthermore, the identification of novel affected biological functions and pathways further shows the added value of the meta-analysis approach.

### Comparison between *E. coli *and *S. aureus *infections

To better evaluate the pathogen-specific characteristics, we further compared the responses to infection with *E. coli *(experiment 1A) or *S. aureus *(experiments 1B and 1C) in the cattle host. We excluded the *S. uberis *data (experiment 2) as we had only one single time point {9} available.

We used the PAMR package to identify the genes which were most dissimilar in terms of their activation in response to the two different pathogens. Of the retained 34 most dissimilar genes, 21 were down-regulated by *E. coli *infection and up-regulated by *S. aureus *infection, while 13 showed the opposite trend (Table 3).

**Table 3:** Dissimilar genes between *E. coli *and *S. aureus *infections in cattle

Gene	Gene Name	*E. coli *shrunken centroid	*S. aureus *shrunken centroid
*ABCG2*	ATP-binding cassette, sub-family G WHITE, member 2	-1.007	0.671
*IDH1*	Isocitrate dehydrogenase 1 NADP+, soluble	-0.929	0.619
*AGPAT1*	1-acylglycerol-3-phosphate O-acyltransferase 1 lysophosphatidic acid acyltransferase, alpha	-0.894	0.596
*PCGF1*	Polycomb group ring finger 1	-0.795	0.53
*GALNTL4*	UDP-N-acetyl-alpha-D-galactosamine:polypeptide N-acetylgalactosaminyltransferase-like 4	-0.52	0.346
*CD74*	CD74 molecule, major histocompatibility complex, class II invariant chain	-0.496	0.33
*TMEM164*	Transmembrane protein 164	-0.42	0.28
*RHOF*	Ras homolog gene family, member F	-0.391	0.261
*MFSD4*	Major facilitator superfamily domain containing 4	-0.263	0.175
*DGCR2*	DiGeorge syndrome critical region gene 2	-0.217	0.145
*FEZ1*	Fasciculation and elongation protein zeta 1 zygin I	-0.204	0.136
*PAOX*	Polyamine oxidase exo-N4-amino	-0.154	0.103
*PMEPA1*	Prostate transmembrane protein, androgen induced 1	-0.106	0.07
*HIGD1B*	HIG1 hypoxia inducible domain family, member 1B	-0.134	0.089
*DNAJC12*	DnaJ Hsp40 homolog, subfamily C, member 12	-0.132	0.088
*VWF*	Von Willebrand factor	-0.131	0.088
*KIAA1467*	KIAA1467	-0.131	0.087
*SENP2*	SUMO1/sentrin/SMT3 specific peptidase 2	-0.068	0.046
*IGFBP5*	Insulin-like growth factor binding protein 5	-0.06	0.04
*SCP2*	Sterol carrier protein 2	-0.018	0.012
*NPAL2*	NIPA-like domain containing 2	-0.009	0.006

*LRRN3*	Leucine rich repeat neuronal 3	0.732	-0.488
*FKBP5*	FK506 binding protein 5	0.7	-0.466
*SLC38A7*	Solute carrier family 38, member 7	0.641	-0.427
*HSPD1*	Heat shock 60 kDa protein 1 chaperonin	0.56	-0.373
*GLUL*	Glutamate-ammonia ligase glutamine synthetase	0.352	-0.235
*CSDA*	Cold shock domain protein A	0.174	-0.116
*INO80E*	INO80 complex subunit E	0.142	-0.095
*SAT1*	Spermidine/spermine N1-acetyltransferase 1	0.118	-0.079
*PHB*	Prohibitin	0.075	-0.05
*STAT3*	Signal transducer and activator of transcription 3 acute-phase response factor	0.061	-0.04
*MAX*	MYC associated factor X	0.051	-0.034
*BTG1*	B-cell translocation gene 1, anti-proliferative	0.033	-0.022
*LCN2*	Lipocalin 2	0.024	-0.016

List of the 34 most dissimilarly regulated genes identified with the PAMR software, showing opposite fold change responses during *E. coli *and *S. aureus *infections in cattle *in vivo *(experiment 1A, B, and C). For each gene, the PAMR shrunken centroid values (using a threshold parameter of 3.77) for the *E. coli *and the *S. aureus *experiments are reported. Twenty-one of the listed genes were up-regulated during infection with *S. aureus*, while 13 were up-regulated during *E. coli *infection.

This list of dissimilar genes was further analyzed with IPA to identify altered biological functions and networks. The 5 most significant molecular and cellular functions identified were cellular development, cellular growth and proliferation, cellular function and maintenance, cell death, and lipid metabolism [Additional file 7]. Both cell death and lipid metabolism were previously found to be among the 5 most significant molecular functions altered in proteins of cows infected with either *E. coli *or *S. aureus *[39]. The IPA network called "antigen presentation, inflammatory response, cell-to-cell signaling and interaction" was the most significantly represented by the list of dissimilar genes. Of the 34 genes, 9 are included in this network: *BTG1*, *CD74, CSDA*, *FKBP5*, *IGFBP5*, *GLUL*, *HSPD1*, *LCN2*, and *PHB*. *IGFBP5 *and *CD74 *were up-regulated after *E. coli *infection and down-regulated after *S. aureus *infection, while the others showed the opposite trend (Table 3).

Pathogen-dependent differences in the time kinetics of induced receptors and defense molecules (e.g. *TLR2*, *TLR4*, *IL-8*, *TNF*, and *NFkB*), as measured by real-time PCR, have been reported between *E. coli *and *S. aureus *[27,55]. Although none of these defense genes were in the list of the 34 most dissimilar genes, our results were in general agreement with these findings as we found that the majority of genes with opposed regulation were associated with immune response and mainly belonged to the antigen presentation, inflammatory response, cell-to-cell signaling and interaction network.

These findings suggest that, at least at the transcriptomic level, these two pathogens cause distinct forms of mastitis infection by the differential modulation of genes belonging to similar molecular pathways and biological functions.

### Comparisons of the 4 lists (I - IV) of affected genes

**Figure 3:**
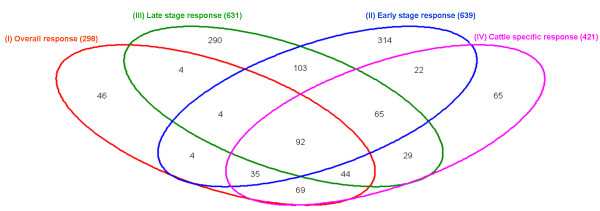
**Venn diagram showing the number of common and combination-specific affected genes**. Venn diagram illustrating the number of significantly affected genes in common (92) and distinct for the four meta-analysis combinations (red: 298 genes of the overall response, green: 631 genes of the late stage response, blue: 639 genes of the early stage response, and pink: 421 genes of the cattle-specific response). The lists of corresponding genes can be found in [Additional file 8].

In order to have an accurate global view of the lists of genes belonging to the 4 different responses to mastitis infection (I to IV), we drew a Venn diagram (Figure 3) that provides a graphical representation of the number of affected genes, as inferred by Pointillist, that are in common, exclusive, or at the various intersections between 2 or 3 lists. The corresponding gene lists with the gene names can be found in [Additional file 8]. Interestingly, we identified a family of antimicrobial genes (*S100A11*, *S100A12*, *S100A8*, and *S100A9*) that were affected in all but the early stage response. This finding was in line with a recent study in cattle, where microarray analysis using Affymetrix gene chip revealed that these genes were differentially expressed after 24 h, but not 6 h, of *E. coli *infection [52].

However, the vast majority of the listed genes have not previously been reported to be implicated in the mastitis infection process. Of particular interest are those genes, a total of 92 [Additional file 8], in common between the 4 (overall, early stage, late stage, and cattle-specific) responses to mastitis (Figure 3), providing possible clues for valuable candidate biomarkers.

### Altered pathways and biological functions related to the 92 genes in common for all 4 responses

The 3 most affected canonical pathways underlying these 92 common genes [Additional file 2] were polyamine regulation, protein ubiquitination, and molecular mechanisms of cancer. The pathways LXR/RXR activation and factors promoting cardiogenesis in vertebrates only approached statistical significance (0.05 < p < 0.1). Altered molecular and cellular functions identified by IPA [Additional file 3] showed general cell related functions (cellular function and maintenance, cellular growth and proliferation, cellular movement, and nucleic acid metabolism) as well as, once again, lipid metabolism.

## Conclusions

To the best of our knowledge this study is the first that statistically combines heterogeneous microarray data realized with different ruminant host species and infected with different mastitis-causing pathogens. The results reinforced previous findings but also revealed several novel themes, including the involvement of genes and pathways that were not identified in individual studies.

Among the 5 most significant molecular and cellular functions common to all 4 gene lists of differential responses to mastitis were cell death, cellular movement, and cellular growth and proliferation, i.e. functions which are intrinsic to general disease response. This indicates that the described procedure of meta-analysis could cope well with the high heterogeneity of the biological systems and the different microarrays used. Indeed, this was confirmed by the analysis of the reduced list of 92 genes in common to all lists that also identified cellular growth and proliferation and cellular movement as being altered.

The results show that protein ubiquitination and polyamine regulation, two pathways involved in immune response modulation and represented by different individual genes, possibly represent a common biological manifestation during mastitis infection in different biological systems. Furthermore, strong complementarities between the early stage and late stages of infection was found, showing a prominence of metabolic and stress signals in the early stage and of the immune response related to the lipid metabolism in the late stage, Both mechanisms were apparently triggered by a small number of genes, including *XBP1 *and *SREBF1*. The cattle-specific response showed an intensification of the immune and inflammatory responses through T lymphocyte involvement. Furthermore, we found several strands of evidence suggesting a correlation between immune response and lipid metabolism as a hallmark of the response of ruminants against mastitis infection.

Overall, the reported meta-analysis approach successfully combined heterogeneous data sets and extracted information of value from individual microarray studies of limited size and statistical power. As such, it provides a global transcriptomic reference which could be useful for the development of novel therapeutics and vaccines for mastitis in ruminants. Furthermore, these data and methodology provide an interesting proof of principle for future studies combining information from diverse sources.

## Methods

### Collection and analysis of microarray data

Microarray data on host responses to infection by mastitis-causing pathogens for various challenge systems were selected to represent contrasting pathogens, hosts, challenge systems (i.e. host tissues or cells, *in vivo *and *in vitro*), sample sizes, time period of observations, microarrays, and signs of infection (summarized in Table 1 with the corresponding references). The experiments were performed with the approval of appropriate ethics committees. Experiment 1 was conducted under the approval of the ethics committee of the regional government in Hannover, Germany (No 509.6-42502-03/678). Experiment 2 was approved by the ethical committee of the Central Veterinary Institute of Wageningen UR in accordance with the Dutch law on animal experiments (registered under number 870.474.05.00.01). Experiment 3 only involved bleeding bovine heifers for 300 ml blood. According to Norwegian legislation no special approval was necessary. The experiments 4 and 6 were, according to the Italian legislation, successfully notified and hence approved by the Italian ethics committee. In experiment 5 ewes were sacrificed in accordance with local regulations (agreement number 31-2010-67) and the study was approved by the INRA animal ethics committee (France).

Spot analysis and quality control of the microarray data for all experiments were done with BlueFuse version 3.1 (BlueGnome, Cambridge, UK; http://www.cambridgebluegnome.com), except for experiment 5 (dendritic cells, DCs) in sheep which was analyzed using SAS ANOVA. The Bioconductor package Limma (Linear Models for Microarray Analysis) in R was used for data normalization and differential expression analyses, comparing gene expression at given times after infection with gene expression in non-infected controls. For each of these analyses p-values were assigned to all genes, indicating the probability that the observed difference in expression occurred by chance. These p-values were then used for the meta-analysis using the Pointillist software. Fold change differences were also calculated and used for specific analyses, in particular for the cattle-specific response.

### Meta-analysis procedures

The 6 datasets from the three ruminant species were obtained from different bovine microarrays, including cDNA arrays (ARK-genomics and National Bovine Functional Genomics Consortium, NBFGC) and commercial oligonucleotide arrays containing 43,768 unique probes (CombiMatrix CustomArray^®^, CombiMatrix Corporation, Seattle, WA, USA) (Table 1).

The preprocessing of the ARK-genomics array data entailed two noteworthy clone ID mapping steps to obtain clone ID consistency throughout all ARK-genomics datasets: the mapping of the clone IDs of a 17K array design onto those of a 20K array design and the mapping of child clone IDs onto the corresponding master clone IDs. The p-values of all groups of master and child clones were averaged, to obtain one value for each master clone ID. Further, the control probes were left out of the meta-analysis, as this was also done for the data stemming from the other microarray platforms.

To compare the probes of the ARK-genomics arrays to those of goat experiment 4 (NBFGC bovine cDNA array, [57]) or goat experiment 6 (CombiMatrix array), a blast comparison between all the spotted sequences was performed. A contiguous perfect match segment of 100 nucleotides (nt) was considered sufficient for probes to be similar. This is a conservative threshold, since perfect matching segments of 30 nt can already cause cross-hybridization in cDNA microarray experiments [58] and since according to the Baldino formula [59] 100 nt long segments under standard conditions can still hybridize while having a mismatch of 15%. A total of 8,302 and 8,293 probes, respectively, were found to be in common.

After evaluation of different meta-analysis methods and programs, an appropriate statistical program called Pointillist (http://magnet.systemsbiology.net/software/Pointillist; [20,21]) that allowed us to account for the relevant experimental differences and the heterogeneity of the datasets, was used to perform meta-analysis. Pointillist is a general-purpose tool that predicts whether system elements are affected by a system perturbation, by integrating different items of evidence of that perturbation. The evidence contains p-values for each addressed element, can address different subsets of the system's elements and may be derived from any type of experiment. In our case the elements are the microarray clones and the items of evidence are the differential expression analyses carried out for selected time points. In a first step Pointillist classifies elements as "affected", if for any of the items of evidence the quantile value of the element's p-value is below a chosen threshold alpha (0.05 in our case). "Combined effective significances (CES)" are calculated by weighting, normalizing, transforming, and combining the element's specific p-values into one single element significance using a Fisher-like transformation (with the Pointillist option called "power") and by finally smoothing the distribution of these significances using a smoothed Gaussian kernel density function. In each step the overlap between the "combined effective significance" distribution for the group of affected and for the group of non-affected elements is iteratively minimized. This process, which is an alternative method to the FDR-adjustment commonly used in the analysis of single data sets, ultimately minimizes the number of false positives and false negatives. The weights used during the transforming operation are also calculated for each item of evidence in each iteration step by comparing the current classification in affected and non-affected elements with the p-value distribution of that evidence. Every Pointillist run contained a row for each probe having a p-value in at least two of the included time points. A special scenario was followed for the final 3-step Pointillist run of the overall analysis, in which the probes common to the cattle and sheep were combined with the probes used in the goat experiments.

### Probe annotation

A probe annotation was performed to transform the microarray probe IDs into gene IDs recognized by Ingenuity Pathways Knowledge Base (IPA, Ingenuity Systems, Mountain View, CA; http://www.ingenuity.com). The annotation started from the probes' EMBL or GenBank accession: the ARK-genomic and CombiMatrix arrays contained probes with references to EMBL accessions in the arrays' GAL files, while the NBFGC array probe names contained references to Genbank accessions. Several probes spotted on the arrays did not have any accession reference due to the incomplete information available at the time of microarray construction. In case these had protein-like names, they were presented as such to IPA. Otherwise they had to be discarded from further analyses. For the probes having an accession reference, an automated stepwise annotation was performed with an in-house script based on sigReannot [60] which took advantage of the recent re-annotation of the cattle genome [61]. A first step verified whether the probes were known to be situated within genomic regions of genes in the Ensembl bovine database (version 52). If this was not the case, in a second step the extracted EMBL or GenBank sequences were mapped to the Ensembl bovine transcripts with a blast cutoff threshold of e-10. In a final step, still unmatched clones were mapped to the complete RefSeq RNA database at NCBI http://www.ncbi.nlm.nih.gov/projects/RefSeq with a blast cutoff threshold of e-5. When the probe coordinates were found to overlap with more than one gene or when blasting against the Ensembl bovine database returned multiple blast hits with a difference in nucleotide coverage between the first and second best hit of <10%, the probe was discarded. For multiple blast hits against the Ensembl bovine database with higher coverage differences, the best covering BLAST hit was nevertheless retained. Next, the Ensembl gene IDs were themselves mapped onto entries from several other target gene databases. For a mapped entry to become the final probe annotation fed to IPA, it obviously had to be recognized by IPA. An arbitrary preference order of the target gene databases was used when screening for IPA recognition: human HGNC, human Entrez, RefSeq Protein, RefSeq RNA, bovine Unigene and bovine Entrez. Also, preference was given to one-to-one mappings.

### Assignment of affected genes to pathways, networks and biological functions

Each gene symbol of the affected genes identified with Pointillist was mapped to its corresponding gene object in the Ingenuity Pathways Knowledge Base. Feeding the aforementioned lists of affected genes as input to the IPA library, significantly associated canonical pathways, biological functions and networks were identified in order to gain biological context and understanding.

Affected biological functions included the sub-groups "Diseases and disorders", "Physiological system development and function" and "Molecular and cellular functions". While the two first sub-groups are highly linked to human diseases and physiology and IPA mainly relies on human data, the third sub-group is relatively general and was better suited for our meta-analysis data. In order to summarize and reduce the vast amount of data generated, which is reported in [Additional files 2 and 3], we focused and discussed in the text the 5 most affected pathways and the 5 most affected biological functions belonging to the sub-group "Molecular and cellular functions".

The found IPA library items were ranked based on significance of association with the input list of genes. For the canonical pathways this significance was determined based on two parameters: (a) ratio of the number of genes from the input data set that map to the canonical pathway divided by the total number of genes of that pathway and (b) p-values calculated using Fischer's exact test determining the probability that the association is explained by chance alone. For the biological functions and networks the significance was linked to the p-value only, calculated by right-tailed Fisher's exact test. The p-values for the network analysis take into account the number of affected genes in the network and the size of the network. Identified networks are presented as a graph indicating the molecular relationships between genes/gene products. Genes are represented as nodes, and the biological relationship between two nodes is represented as an edge (line). All edges are supported by at least one reference from the literature, from a textbook, or from information stored in the IPA Knowledge Base. The intensity of the node color indicates the degree of up- (red) or down-regulation (green). Genes in uncolored nodes were not identified as differentially expressed in the experiment. The intrinsic size of networks, functions and pathways, used in the calculation of the significance of association, depend on the chosen IPA gene "universe". We did not change the IPA default "universe", basically containing all genes and endogenous chemicals of the IPA library.

The additional IPA function called "building pathway" was used to graphically show the relationship and interactions between genes belonging to significantly affected IPA gene networks during the early stage response to mastitis, and to connect all lipogenic genes identified during the late stage response.

### Venn diagram and heat map building, and visualization of fold change variations in different cattle experiments

The Venn diagram was built using R script overLapper.R http://faculty.ucr.edu/~tgirke/Documents/R_BioCond/My_R_Scripts/overLapper.R.

The heat map was constructed with the "heatmap" function of the R package "stats". The R package MaSigPro [53] was used to visualize the magnitude of fold change expressions during the time course of the different cattle microarray experiments 1A {time points 1, 2, 3}, 1B {time points 4, 5, 6}, 1C {time points 7, 8}, 2 {time point 9}, and 3 {time points 10, 11}.

### Fold change dissimilarities between *E. coli *and *S. aureus *infections in cattle *in vivo*

The R package PAMR was used to detect dissimilarities among fold change responses to *E. coli *and *S. aureus *pathogen infections *in vivo *in cattle (experiment 1A, 1B, and 1C, Table 1). The PAMR algorithm performs an expression-profile based sample class prediction [62]. In a first step, average within-class expression profiles, so called "centroids", are calculated for all sample classes. In a next step, these centroids are shrunken, shifting the average within-class expression of each gene towards the gene's overall expression average, and taking a gene out of the centroid when its within-class expression average coincides with the overall one. The extent of gene expression shrinkage is proportional to the gene's within-class standard deviation, and is also determined by the chosen "threshold" or "shrinkage" parameter. The higher the threshold, the fewer genes that are retained in the class shrunken centroids and the more dissimilar they are. Finally, samples can then be classified by mapping them to the shrunken centroid that is nearest to the sample's expression profile. Here we used PAMR to construct shrunken centroids of the two classes of the *E. coli *and *S. aureus *infected samples. For a range of threshold parameters, PAMR evaluated the classification accuracy and the size of the resulting shrunken centroids. Out of the threshold parameters yielding the highest classification accuracy, we selected the lowest threshold parameter that brought the shrunken centroid's size below an arbitrarily chosen limit of 50 dissimilar genes. In this specific case, a threshold parameter of 3.77 was selected, and this resulted in the 34 most dissimilar genes being retained in the resulting shrunken centroids. These dissimilar genes were further examined with IPA.

## Authors' contributions

SG, BB, and GS wrote the paper, collated the microarray data, and performed the meta-analysis. SCB helped to conceive the meta-analysis project, supervised the meta-analysis, helped to write the paper and helped to collate the microarray data. DW performed analysis of single microarray experiments and supervised the meta-analysis. MHP coordinated the entire EADGENE project, helped collecting and sharing the microarray data. CC and CK developed bioinformatic tools used to produce the gene annotations for this study. HMS coordinated the work of the EADGENE's groups working on mastitis and developed microarray experiment 1, in particular arranged for the animal infection experiments, sample collection, and initial RNA preparations. WP conducted the animal infections, recorded the zootechnical/health parameters throughout the experiments, and prepared all the tissues. KJ did all the hybridizations and data analysis of experiment 1. EJG developed in conjunction with HMS experiment 1 and helped in sample preparation and data analysis. AdG performed the experimental infection with *S. uberis *and performed microarray experiment 2. HES initiated the mastitis work at the central veterinary institute, wrote the *S. uberis *project, and designed the experimental infection of experiment 2. MAS was the contact person for EADGENE at the Animals Sciences Group and was closely involved in the initiation of the operational genomics work package within the network. IO was involved in the planning of the NSVS microarray experiment 3, partly participated in the practical work, and helped with the analysis of the data and the manuscript writing. GMB conducted the microarray experiment 3 in the laboratory (biological samples preparation and hybridization). PM, GP, BC, and PC organized the experiments, collected the samples, performed the experimental infections with *S. aureus *in goat, and analyzed the microarray data of experiments 4 and 6. MDC prepared and edited the data of experiment 6. EF conducted the microarray experiment 5 in the laboratory (biological samples preparation and hybridization). GF planned the experimental design of microarray experiment 5 in sheep. RR developed the design of microarray experiment 5 and did statistical analysis of the data. EG helped to conceive the meta-analysis project, coordinated the overall project and helped in writing this manuscript. All authors read and approved the final manuscript.

## Supplementary Material

Additional file 1 **Lists of affected genes during different responses to mastitis infection**. Complete lists of affected genes and corresponding "Combined Effective Significances (CES)" identified with pointillist for the 4 main responses to mastitis (I) overall response, (II) early stage response, (III) late stage response, (IV) cattle-specific response, as well as the two additional time dependent responses (V) early specific and (VI) late specific. Click here for file

Additional file 2 **Lists of all affected canonical pathways and corresponding affected genes**. Complete lists of affected canonical pathways (p < 0.05) and corresponding affected genes identified with IPA for the meta-analysis combinations (I) overall response, (II) early stage response, (III) late stage response, (IV) cattle-specific response, (V) early specific response and (VI) late specific response, as well as for the common affected genes between the 4 meta-analysis responses (I) to (IV) (Figure 3, n = 92). The identified canonical pathways are listed from the lowest to the highest p-value. An asterisk indicates that the pathway approached statistical significance (0.05<p < 0.1). Click here for file

Additional file 3 **Lists of all affected biological functions and corresponding affected genes**. Complete lists of all affected biological functions (p < 0.05) and corresponding affected genes identified with IPA for the meta-analysis combinations (I) overall response, (II) early stage response, (III) late stage response, (IV) cattle-specific response, (V) early specific response and (VI) late specific response, as well as for the common affected genes between the 4 meta-analysis responses (I) to (IV) (Figure 3, n = 92). The biological functions include all the sub-groups "Diseases and disorders", "Physiological system development and function" and "Molecular and cellular functions" and are listed from the lowest to the highest p-value. The five most affected molecular and cellular functions, which are discussed in the text, are in bold. Click here for file

Additional file 4 **Affected sub-functions of lipid metabolism during different responses to mastitis infection**. Five most significant sub-functions of lipid metabolism that are altered during (I) overall, (II) early stage, (III) late stage, and (IV) cattle-specific responses. The results were obtained by IPA using the lists of significantly affected genes for each specific response. The sub-functions of the lipid metabolism are listed from the lowest to the highest p-value, and are reported with the involved genes. Click here for file

Additional file 5 **Supplemental Figure S1 - Relationship between *XBP1 *and additional affected genes during the early stage response to mastitis**. Gene network showing the connections, as identified with the IPA option "building pathways", between the gene *XBP1 *and other affected genes during (II) early stage response to mastitis infection. **A**. *XBP1 *is related and linked to several other affected genes. **B**. *XBP1 *is directly linked to the genes *COPZ1*, *DDOST*, *KDELR2*, *KDELR3*, *RPN1*, *SEC23B*, *SEC24D*, *SEC61A1*, and *SRPR*, as well as to genes of the proteasome and the MHC Class II complex. Supplemental Figure S2 - Relationship between *SREBF1 *and additional affected genes during the late stage response to mastitis. Gene network showing the connections, as identified with the IPA option "building pathways", between affected genes involved in lipid metabolism during (III) late stage response to mastitis infection. The gene *SREBF1 *seems to play an important role and is directly linked to other affected genes (violet colour), i.e. *TRAF3IP3*, *CD36*, *SCD*, *SOD1*, *IDH1*, *THRB*, *RETN*, *PMVK*, *DBI*, *UCP2*, *HBS1*, *SC4MOL*, and *CYP27A1*. Supplemental Figure S3 - Venn diagram showing the number of common and experiment-specific affected genes between (IV) cattle-specific response and the individual experiments 1A time point {3} and 2 time point {9}. Venn diagram illustrating the number of significantly affected genes in common (25) and distinct for the (IV) cattle-specific response (red: 421 genes), experiment 1A time point {3} (green: 745 genes), and experiment 2 time point {9} (blue: 55 genes). The lists of corresponding genes can be found in [Additional file 6]. The list of experiments and time points can be found in Table 1 and the list of meta-analysis combinations in Table 2. Click here for file

Additional file 6 **Lists of affected genes that are distinct or in common between (IV) cattle-specific response, experiment 1A time point {3}, and experiment 2 time point {9}**. Complete lists of affected genes corresponding to the Venn diagram [Additional file 5: Supplemental Figure S3], including genes that are distinct or in common at the intersections between (IV) cattle-specific response, experiment 1A time point {3}, and experiment 2 time point {9}. The list of experiments and time points can be found in Table 1 and the list of meta-analysis combinations in Table 2. Click here for file

Additional file 7 **Affected molecular and cellular functions of the most dissimilar genes between *E. coli *and *S. aureus*.** Five most significant molecular and cellular functions identified with IPA using the 34 most dissimilar genes between *E. coli *and *S. aureus *infections in cattle *in vivo *(experiment 1A, 1B, and 1C), as found with the PAMR software (Table 3). The identified molecular and cellular functions are listed from the lowest to the highest p-value, and are reported with the involved genes. Click here for file

Additional file 8 **Lists of affected genes that are distinct or in common between the 4 main responses to mastitis infection**. Complete lists of affected genes corresponding to the Venn diagram in Figure 3, including genes that are distinct or in common at the intersections between the 4 different responses (I) overall, (II) early stage, (III) late stage, and (IV) cattle-specific. Click here for file
