# Supplementary material for: Strengthening insights into host responses to mastitis infection in ruminants by combining heterogeneous microarray data sources
Source: BMC Genomics. 2011 May 11;12:225. doi: 10.1186/1471-2164-12-225 (PMC3118214; doi:10.1186/1471-2164-12-225)
Supplement: Additional file 4 — Affected sub-functions of lipid metabolism during different responses to mastitis infection. Five most significant sub-functions of lipid metabolism that are altered during (I) overall, (II) early stage, (III) late stage, and (IV) cattle-specific responses. The results were obtained by IPA using the lists of significantly affected genes for each specific response. The sub-functions of the lipid metabolism are listed from the lowest to the highest p-value, and are reported with the involved genes. [file 1471-2164-12-225-S4.DOC]

**Additional file 4 – Affected sub-functions of lipid metabolism during different responses to mastitis infection**

Five most significant sub-functions of lipid metabolism that are altered during (I) overall, (II) early stage, (III) late stage, and (IV) cattle-specific responses. The results were obtained by IPA using the lists of significantly affected genes for each specific response. The sub-functions of the lipid metabolism are listed from the lowest to the highest p-value, and are reported with the involved genes.

| **Sub-function of lipid metabolism** | **Genes** | **P-value** |
| --- | --- | --- |
| **(I) Overall response** |  |  |
| Metabolism of long chain fatty acid | *ACSL6, CD36, SLC27A2, SLC27A5* | 1.08E-04 |
| Accumulation of oleic acid | *CD36, SCD* | 7.86E-04 |
| Internalization of lipid | *ACSL6, CAV1, TSPO* | 8.46E-04 |
| Uptake of fatty acid | *CD36, S100A8, S100A9, SLC27A2, SLC27A5* | 1.07E-03 |
| Uptake of arachidonic acid | *S100A8, S100A9* | 2.56E-03 |
|  |  |  |
| **(II) Early stage response** |  |  |
| Hydrolysis of phosphatidylinositol phosphate | *INPP5K, TMEM55A, TMEM55B* | 3.48E-04 |
| Hydrolysis of phosphatidylinositol 4,5-diphosphate | *AGT, INPP5K, PLCG2, TMEM55A, TMEM55B* | 1.00E-03 |
| Hydrolysis of phosphtidylinositol 5-phosphate | *TMEM55A, TMEM55B* | 3.24E-03 |
| Metabolism of fatty acid | *ACAA1, ACOT7, ACOT8, ACOT9, ACSL6, AGT, CD36, FAAH, HMGCL, HSD17B4, PDK4, SCP2, SLC27A5* | 5.68E-04 |
| Metabolism of lipids | *ACAA1, ACOT7, ACOT8, ACOT9, ACSL6, AGPAT1, AGT, ALDH3A2, APOE, APOH, CD36, CD74, CHPT1, CYP1B1, ETNK2, FAAH, GM2A, HMGCL, HSD17B4, LPIN1, LRP5, PDK4, PLCG2, PLD1, PMVK, PPAP2B, SCP2, SLC27A5* | 4.14E-03 |
|  |  |  |
| **(III) Late stage response** |  |  |
| Quantity of fatty acid | *APEX1, APOE, CAV1, CD36, CYP27A1, FABP3, GNAS, GPX1, IDH1, MYC, RETN, SAT1, SCD, SCP2, SOD1, SREBF1, XDH* | 3.11E-06 |
| Quantity of oleic acid | *CYP27A1, SCD, SREBF1* | 4.23E-05 |
| Quantity of lipid | *ABCA3, ABCG2, APEX1, APOE, B4GALT1, CAV1, CD36, CYP27A1, EEF1A2, FABP3, FCER1G, GNAS, GPX1, HIF1A, IDH1, IGF2, IL1RN, MAPKAPK2, MYC, PLD1, PPAP2B, RETN, RHOA, SAT1, SCD, SCP2, SOD1, SREBF1, STAT3, UCP2, XDH* | 6.46E-04 |
| Synthesis of lipid | *AGPAT1, APOE, B4GALT1, C14ORF147, CD9, CD14, CD74, CHKB, CHPT1, CYP27A1, FABP3, FCER1G, GPR77, HEXB, IGF2, LASS2, LYN, PLAU, PLD1, PTAFR, PTGES3, PTX3, RHOA, SCD, SCP2, SOD1, SREBF1* | 4.47E-05 |
| Synthesis of cholesterol | *APOE, CAV1, CYP27A1, DHCR24, MVK, SCP2, SREBF1* | 1.86E-03 |
|  |  |  |
| **(IV) Cattle-specific response** |  |  |
| Uptake of arachidonic acid | *S100A8, S100A9, SCARB1* | 4.88E-05 |
| Metabolism of long chain fatty acid | *ACSL6, CD36, SLC27A2, SLC27A5* | 4.17E-04 |
| Internalization of cholesterol | *SCARB1, TSPO* | 5.38E-04 |
| Transport of fatty acid | *ABCG2, ACSL6, ALB, CD36, GOT2, SCP2* | 7.22E-04 |
| Quantity of fatty acid | *APEX1, CAV1, CD36, IDH1, MYC, SAT1, SCARB1, SCD, SCP2, SOD1* | 9.07E-04 |
