# Supplementary material for: Strengthening insights into host responses to mastitis infection in ruminants by combining heterogeneous microarray data sources
Source: BMC Genomics. 2011 May 11;12:225. doi: 10.1186/1471-2164-12-225 (PMC3118214; doi:10.1186/1471-2164-12-225)
Supplement: Additional file 5 — Supplemental Figure S1 - Relationship between XBP1 and additional affected genes during the early stage response to mastitis. Gene network showing the connections, as identified with the IPA option "building pathways", between the gene XBP1 and other affected genes during (II) early stage response to mastitis infection. A. XBP1 is related and linked to several other affected genes. B. XBP1 is directly linked to the genes COPZ1, DDOST, KDELR2, KDELR3, RPN1, SEC23B, SEC24D, SEC61A1, and SRPR, as well as to genes of the proteasome and the MHC Class II complex. Supplemental Figure S2 - Relationship between SREBF1 and additional affected genes during the late stage response to mastitis. Gene network showing the connections, as identified with the IPA option "building pathways", between affected genes involved in lipid metabolism during (III) late stage response to mastitis infection. The gene SREBF1 seems to play an important role and is directly linked to other affected genes (violet colour), i.e. TRAF3IP3, CD36, SCD, SOD1, IDH1, THRB, RETN, PMVK, DBI, UCP2, HBS1, SC4MOL, and CYP27A1. Supplemental Figure S3 - Venn diagram showing the number of common and experiment-specific affected genes between (IV) cattle-specific response and the individual experiments 1A time point {3} and 2 time point {9}. Venn diagram illustrating the number of significantly affected genes in common (25) and distinct for the (IV) cattle-specific response (red: 421 genes), experiment 1A time point {3} (green: 745 genes), and experiment 2 time point {9} (blue: 55 genes). The lists of corresponding genes can be found in [Additional file 6]. The list of experiments and time points can be found in Table 1 and the list of meta-analysis combinations in Table 2. [file 1471-2164-12-225-S5.DOC]

**Additional file 5: Supplemental Figure S1 – Relationship between *XBP1* and additional affected genes during the early stage response to mastitis**

Gene network showing the connections, as identified with the IPA option "building pathways", between the gene *XBP1* and other affected genes during (II) early stage response to mastitis infection. **A**. *XBP1* is related and linked to several other affected genes. **B**. *XBP1* is directly linked to the genes *COPZ1*, *DDOST*, *KDELR2*, *KDELR3*, *RPN1*, *SEC23B*, *SEC24D*, *SEC61A1*, and *SRPR*, as well as to genes of the proteasome and the MHC Class II complex.

**A**.
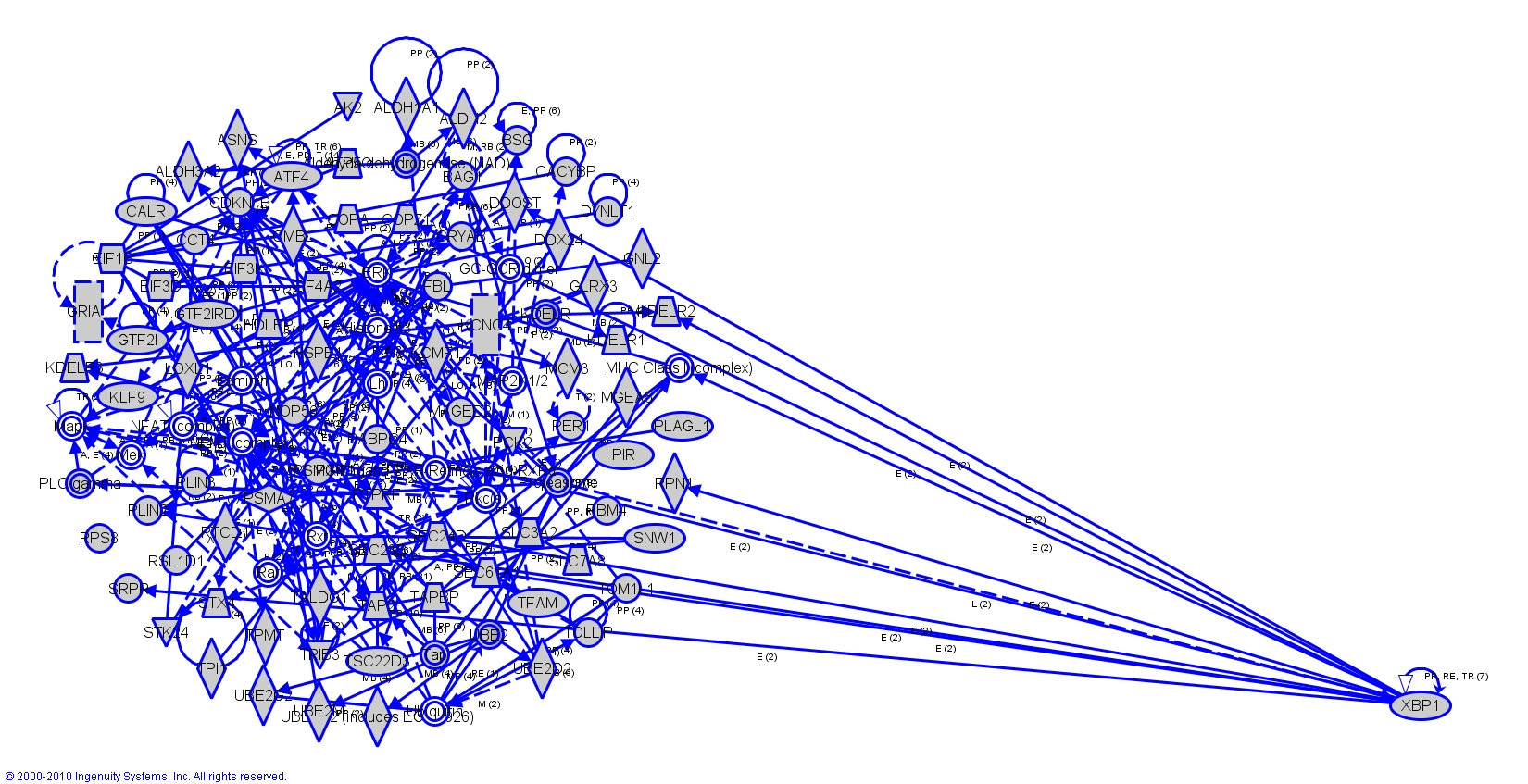


**B**.


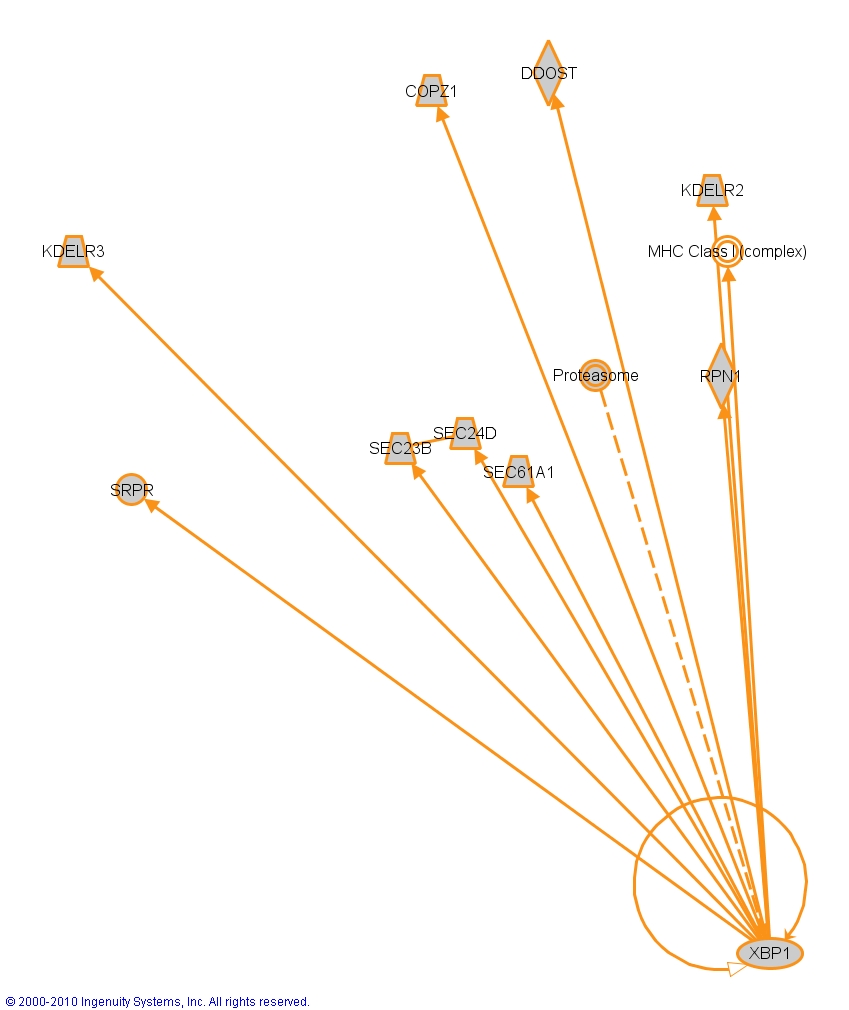


**Additional file 5: Supplemental Figure S2 – Relationship between *SREBF1* and additional affected genes during the late stage response to mastitis**

Gene network showing the connections, as identified with the IPA option "building pathways", between affected genes involved in lipid metabolism during (III) late stage response to mastitis infection. The gene *SREBF1* seems to play an important role and is directly linked to other affected genes (violet colour), i.e. *TRAF3IP3*, *CD36*, *SCD*, *SOD1*, *IDH1*, *THRB*, *RETN*, *PMVK*, *DBI*, *UCP2*, *HBS1*, *SC4MOL*, and *CYP27A1*.


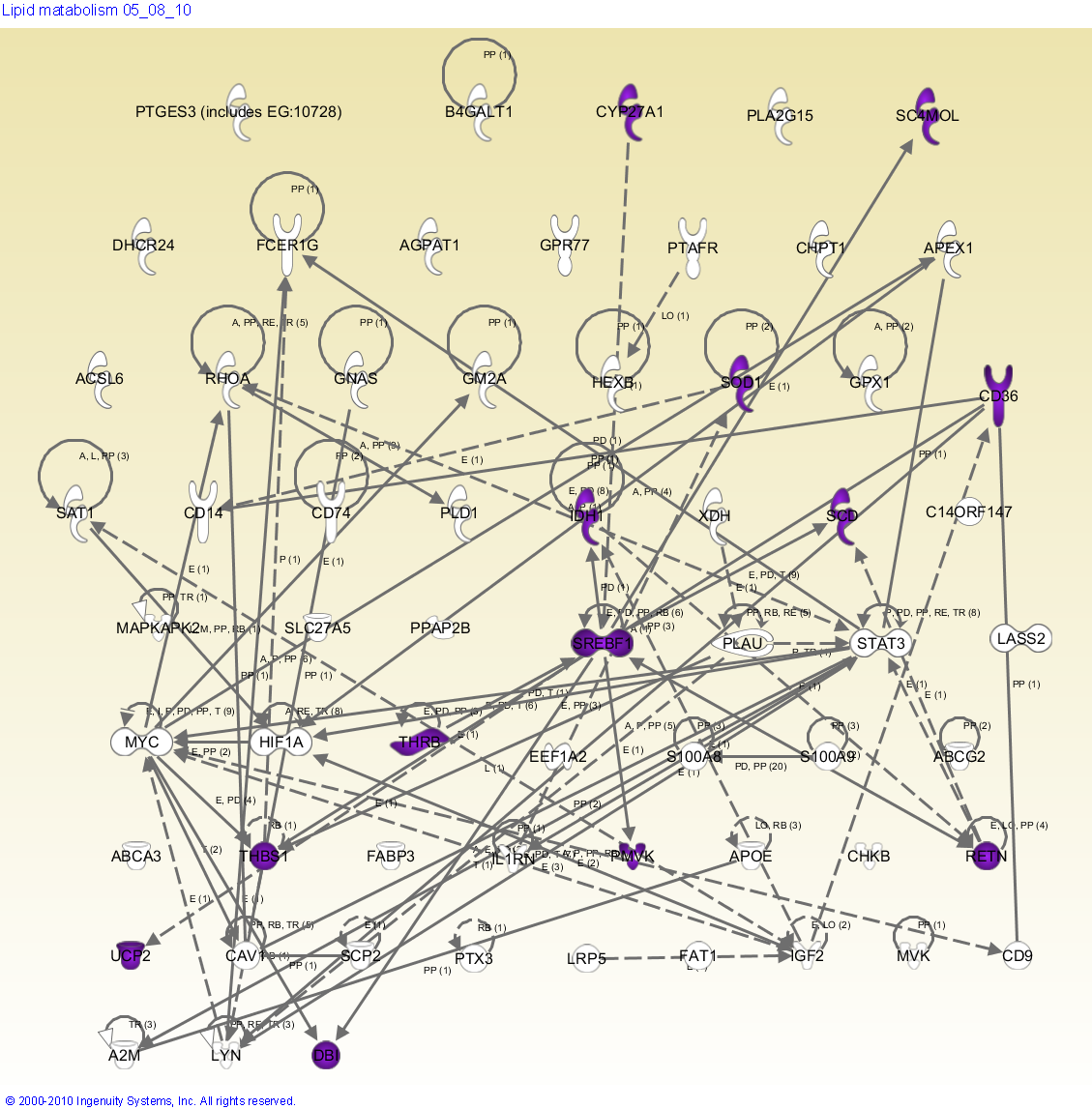


**Additional file 5: Supplemental Figure S3 – Venn diagram showing the number of common and experiment-specific affected genes between (IV) cattle-specific response and the individual experiments 1A time point {3} and 2 time point {9}**

Venn diagram illustrating the number of significantly affected genes in common (25) and distinct for the (IV) cattle-specific response (red: 421 genes), experiment 1A time point {3} (green: 745 genes), and experiment 2 time point {9} (blue: 55 genes). The lists of corresponding genes can be found in [Additional file 6]. The list of experiments and time points can be found in Table 1 and the list of meta-analysis combinations in Table 2.


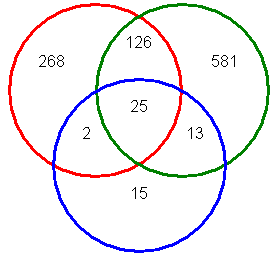


**(IV) cattle-specific response (421)**

**experiment 1A time point {3} (745)**

**experiment 2 time point {9} (55)**
