# Supplementary material for: Strengthening insights into host responses to mastitis infection in ruminants by combining heterogeneous microarray data sources
Source: BMC Genomics. 2011 May 11;12:225. doi: 10.1186/1471-2164-12-225 (PMC3118214; doi:10.1186/1471-2164-12-225)
Supplement: Additional file 7 — Affected molecular and cellular functions of the most dissimilar genes between E. coli and S. aureus. Five most significant molecular and cellular functions identified with IPA using the 34 most dissimilar genes between E. coli and S. aureus infections in cattle in vivo (experiment 1A, 1B, and 1C), as found with the PAMR software (Table 3). The identified molecular and cellular functions are listed from the lowest to the highest p-value, and are reported with the involved genes. [file 1471-2164-12-225-S7.DOC]

**Additional file 7 – Affected molecular and cellular functions of the most dissimilar genes between *E. coli* and *S. aureus***

Five most significant molecular and cellular functions identified with IPA using the 34 most dissimilar genes between *E. coli* and *S. aureus* infections in cattle *in vivo* (experiment 1A, 1B, and 1C), as found with the PAMR software (Table 3). The identified molecular and cellular functions are listed from the lowest to the highest p-value, and are reported with the involved genes.

| **IPA molecular and cellular functions** | **Genes** | **P-value** |
| --- | --- | --- |
| Cellular development | *ABCG2, BTG1, CD74, CSDA, FEZ1, FKBP5, GLUL, HSPD1, IGFBP5, LCN2, MAX, PMEPA1, SAT1, STAT3, VWF* | 2.24E-06 |
| Cellular growth and proliferation | *ABCG2, BTG1, CD74, CSDA, FKBP5, HSPD1*, *IGFBP5, LCN2, MAX, PHB, PMEPA1, SAT1, STAT3* | 2.24E-06 |
| Cellular function and maintenance | *ABCG2, CD74, HSPD1, SAT1, STAT3* | 8.78E-06 |
| Cell death | *ABCG2, BTG1, CD74, CSDA, FKBP5, HSPD1, IGFBP5, LCN2, MAX, PHB, PMEPA1, SAT1, SCP2, STAT3* | 1.10E-05 |
| Lipid metabolism | *ABCG2, AGPAT1, CD74, FKBP5, HSPD1, IDH1, SAT1, SCP2, STAT3* | 4.37E-05 |
